# Changes in oral flora of patients with functional dyspepsia

**DOI:** 10.1038/s41598-021-87600-5

**Published:** 2021-04-13

**Authors:** Xu-juan Liu, Wen-rui Xie, Li-hao Wu, Zhi-ning Ye, Xue-yuan Zhang, Ran Zhang, Xing-xiang He

**Affiliations:** 1 Department of Gastroenterology, The First Affiliated Hospital of Guangdong Pharmaceutical University, NO 19, Nonglinxia Road, Yuexiu District, Guangzhou, 510080 Guangdong Province China; 2 Integrated Hospital of Traditional Chinese, Southern Medical University, Guangzhou, Guangdong China; 3 Research Center for Engineering Techniques Therapies of Guangdong Province, NO 19, Nonglinxia Road, Yuexiu District, Guangzhou, Guangdong Province China

**Keywords:** Microbiology, Biomarkers, Diseases, Gastroenterology, Pathogenesis, Risk factors

## Abstract

To explore the changes in oral flora in people with functional dyspepsia (FD). Unstimulated saliva was collected from 21 FD patients diagnosed according to the Rome IV criteria and from 12 healthy controls (HCs) for 16SrRNA sequencing. The pH of saliva samples and community periodontal index (CPI) were tested. The prevalence of small intestinal bacterial overgrowth (SIBO) was obtained by the methane-and hydrogen-based breath test. At the phylum level, FD patients had a higher relative abundance of Spirochaetes and a lower relative abundance of Fusobacteria*,* TM7 and Proteobacteria than HCs (*p* < 0.01). In the saliva, *Kingella* and *Abiotrophia* genus levels showed significant changes between the FD and HC groups (*p* < 0.01). Salivary species level marker *Intermedia* was significantly different between FD and HC groups (*p* < 0.01). The oral pH of FD patients was higher than that of HCs (*p* < 0.01). The mean CPI of the FD group was 1.52 and that of the HC group was 0.17 (*p* < 0.01). Moreover, 71.4% of the FD group was positive for SIBO. The oral flora of FD patients was different from that of HCs. Spirochaetes, *Kingella, Abiotrophia,* and *Intermedia* may be diagnostic indicators of FD.

## Introduction

Functional dyspepsia (FD) is a symptom-based gastrointestinal disorder characterized by the absence of organic, systemic, or metabolic disease causative of the complaints^1^. FD is associated with significant morbidity, psychological disturbances, overall impaired quality of life, and an increased financial burden for patients^2–9^. However, pathogenesis of FD is largely unknown.

Previous studies have shown that etiology of FD is complex, including many aspects, such as delayed gastric emptying, impaired gastric accommodation to a meal, and autonomic nervous system–central nervous system dysregulation^10–13^. Recent studies have suggested that microbiota dysregulation plays an important role in some functional gastrointestinal disorders^14^. Furthermore, some studies have suggested that alterations of the oral flora are associated not only with periodontal diseases^15^ but also disturbances of the flora in the upper gastrointestinal tract^16^. No study has explored the role of oral flora in the pathogenesis of FD.

We wished to study changes of oral flora in FD and to compare the salivary bacterial levels, oral pH, community periodontal index (CPI), Decay Missed Filled Tooth (DMFT), and small intestinal bacterial overgrowth (SIBO) between patients with FD and healthy controls (HCs).

## Materials and methods

### Ethical approval of the study protocol

The study protocol was approved by the research ethics committee of the First Affiliated Hospital of Guangdong Pharmaceutical University (2018 [86]) in Guangdong, China. All subjects signed the informed consent forms. Our study is registered with the China Clinical Trial Registry (NO ChiCTR1900021994). Written informed consent was obtained from each participant. The consent form included statements that (1) identified the institutional and/or licensing committee approving the experiments, including any relevant details; (2) confirmed that all experiments were performed in accordance with relevant guidelines and regulations.

### Participants

We recruited 21 patients who met the Rome IV criteria for FD^17^. FD patients were recruited from clinics at the First Affiliated Hospital of Guangdong Pharmaceutical University in China.

The exclusion criteria were: (1) known genetic conditions; (2) clinically evident serious infections or inflammatory conditions; (3) history of cancer; (4) intestinal organic disease. Individuals who had received probiotic treatment were asked to stop treatment ≥ 1 week before sample collection, and people were excluded if they had taken antibiotics in the preceding month.

The 12 recruited HCs had to: (1) be in good health; (2) not meet any diagnostic criteria for functional and organic diseases; (3) not have taken drugs that affect oral and intestinal flora in the past month.

### Handling and collection of samples

All participants underwent sequencing of 16SrRNA gene amplicons in saliva samples, salivary pH testing, oral CPI testing, and hydrogen- and methane-based breath testing. The specific sample collection methods and test details are as follows:

#### Sequencing of 16SrRNA gene amplicons in saliva samples

To obtain samples of the oral microbiome, individuals were asked to produce 10 mL of saliva after refraining from eating, drinking, and oral-hygiene practice for 8 h. Samples were collected in sterile DNA- and RNA-free 15-mL centrifuge tubes (Precidiag, Sevres, France) and frozen immediately at − 80 °C. De-identified and coded samples were shipped to Exon Bio (Guangzhou, China) on dry ice for extraction and sequencing of DNA.

#### Salivary pH

Participants were seated comfortably on a chair with their heads bent forward. They were asked to spit into a sterile cup. Saliva was collected after participants had refrained from eating, drinking, and oral-hygiene practice for 8 h. Salivary pH was estimated directly using a digital pH meter (Toledo S220-K SevenCompact pH Meter; Mettler, Geneva, Switzerland) and calibrated against a range of buffers at pH 4.0, 7.0, and 9.21, as described previously^18,19^. Readings were obtained by a recorder blinded to the study protocol. The saliva containers were coded to eliminate the possibility of observer bias.

#### Distribution of oral diseases

Dental evaluations were undertaken by a single trained dentist to avoid biases. Oral examination was validated using the CPI and DMFT^20^. CPI is to examine gingival bleeding, periodontal pocket depth and dental calculus on index teeth. The scoring criteria that we used was: 0 = gum health; 1 = gingivitis, with bleeding after exploratory examination; 2 = calculus can be detected, but the black part of the probe is exposed outside the gingival pocket; 3 = early periodontal disease, the gingival margin covers part of the black part of the probe, and the gingival pocket has a depth of 4–5 mm; 4 = advanced periodontal disease, the black part of the probe is completely covered by the gingival margin, and the depth of the periodontal pocket is > 6 mm; X = excluded segments (< 2 functional teeth); 9 = unable to check.

#### Testing of hydrogen and methane in breath

All participants were required to stop using antibiotics, probiotics, lactulose, acid-producing agents, or drugs affecting gastrointestinal dynamics 4 weeks before the examination. An enema had not been given in the past 2 weeks, and a history of acute enteritis was not found. Briefly, 24 h before inspection, participants were instructed to refrain from consuming dairy products, soybean products, wheat, flour products, high-fiber vegetables, and other hydrogen-producing food, and to avoid overeating. Participants were asked to follow a strict, low-residue diet the day before the test and were asked not to eat, drink, or smoke tobacco for the previous 12 h. After obtaining an initial breath sample at baseline, patients ingested 10 g of lactulose dissolved in 50 mL of water. This was followed by measuring the hydrogen and methane in breath samples every 30 min for 150 min^21^. Levels of methane, hydrogen, and carbon dioxide were measured by a Breath Tracker (QuinTron, Santa Maria, CA, USA). Concentrations of hydrogen and methane were corrected for levels of carbon dioxide. Cumulative measurements of hydrogen or methane over time were integrated into a single measurement by the area under the concentration curve (AUC) using the trapezoid method^22^: methane (AUC-CH_4_) and hydrogen (AUC-H_2_).

### Statistical analyses

SPSS v22.0 (IBM, Armonk, NY, USA) was used to analyze data. Clinical and demographic data were compared using the Student’s *t* test or chi-square test as appropriate. Differences in diversity, taxonomic composition, and community function were evaluated using the Kruskal–Wallis or Wilcoxon rank-sum test. A *p* value < 0.05 (two-sided) was considered significant.

## Results

### Demographic and clinical characteristics

Twenty-one FD patients and 12 HCs were enrolled in this study. Evaluation of demographic and clinical characteristics showed no significant differences in age, sex, or body mass index (BMI) in the FD and HC groups (Table 1).

**Table 1:** Comparison of baseline characteristics (mean ± SD).

Characteristics	FD (n = 21)	HC (n = 12)	*p*
Sex			
Male; n (%)	9 (42.9)	7 (58.3)	0.490
Female; n (%)	12 (57.1)	5 (41.7)	
Age (years); $\overline{X}$ ± S	37.96 ± 15.79	25.03 ± 6.17	0.704
BMI; $\overline{X}$ ± S	22.26 ± 1.78	20.48 ± 1.30	0.234
Education years	16.15 ± 2.19	16.08 ± 2.39	0.194

### Sequencing of 16SrRNA gene amplicons

**Figure 1:**
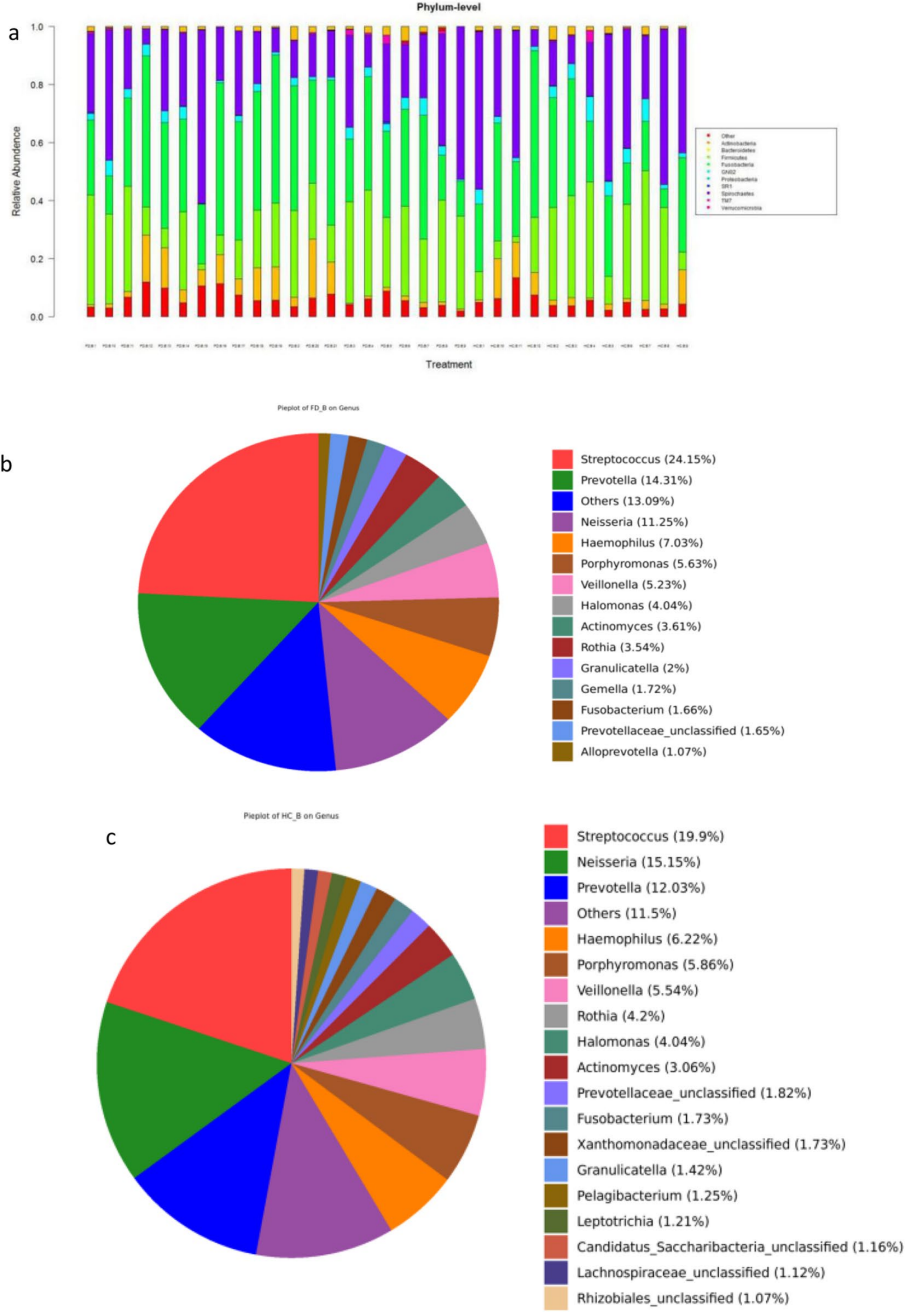

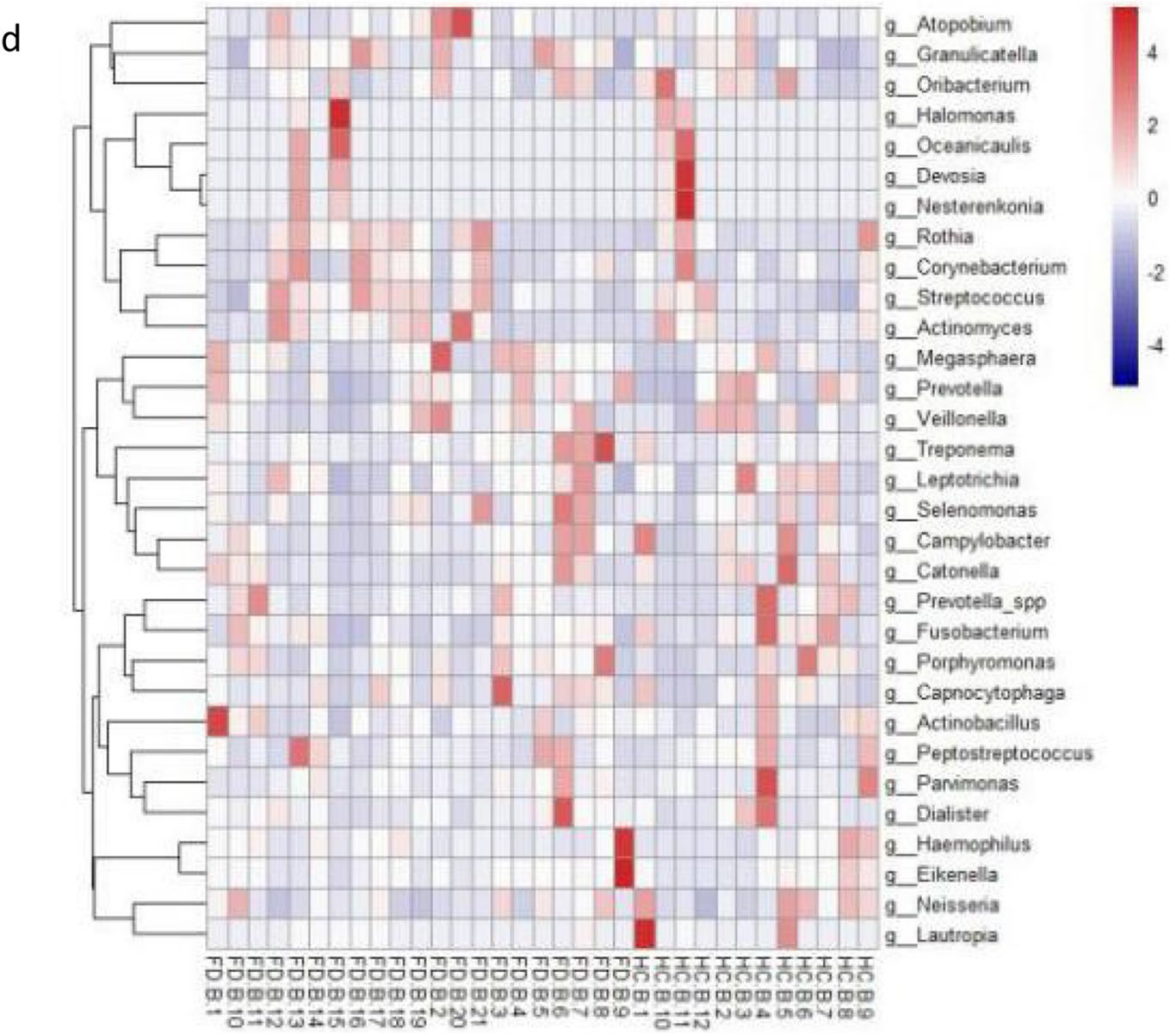
Relative abundance of saliva microbiota in FD patients and HCs. (**a**) Bar plots of relative abundance of phylum-level microbiota in the FD group and HC group. (**b**) Bar plots of relative abundance of genus-level microbiota in the FD group and HC group. (**c**) Relative abundance of genus-level microbiota in HCs. (**d**) Heatmaps showing the expression profile of genus-level microbiota.

At the phylum level, Firmicutes*,* Proteobacteria*,* Bacteroidetes*,* Spirochaetes*,* Actinobacteria*,* Fusobacteria, and TM7 constituted the vast majority of the predominant salivary microbiota (Fig. 1a). The major genera that contributed to the oral flora in the FD and HC groups was *Streptococcus, Prevotella,* and *Neisseria*. Overall, there were significant inter-subject and inter-pair variabilities in microbiota composition (Fig. 1b–d).

### FD subjects harbor an altered oral flora

**Figure 2:**
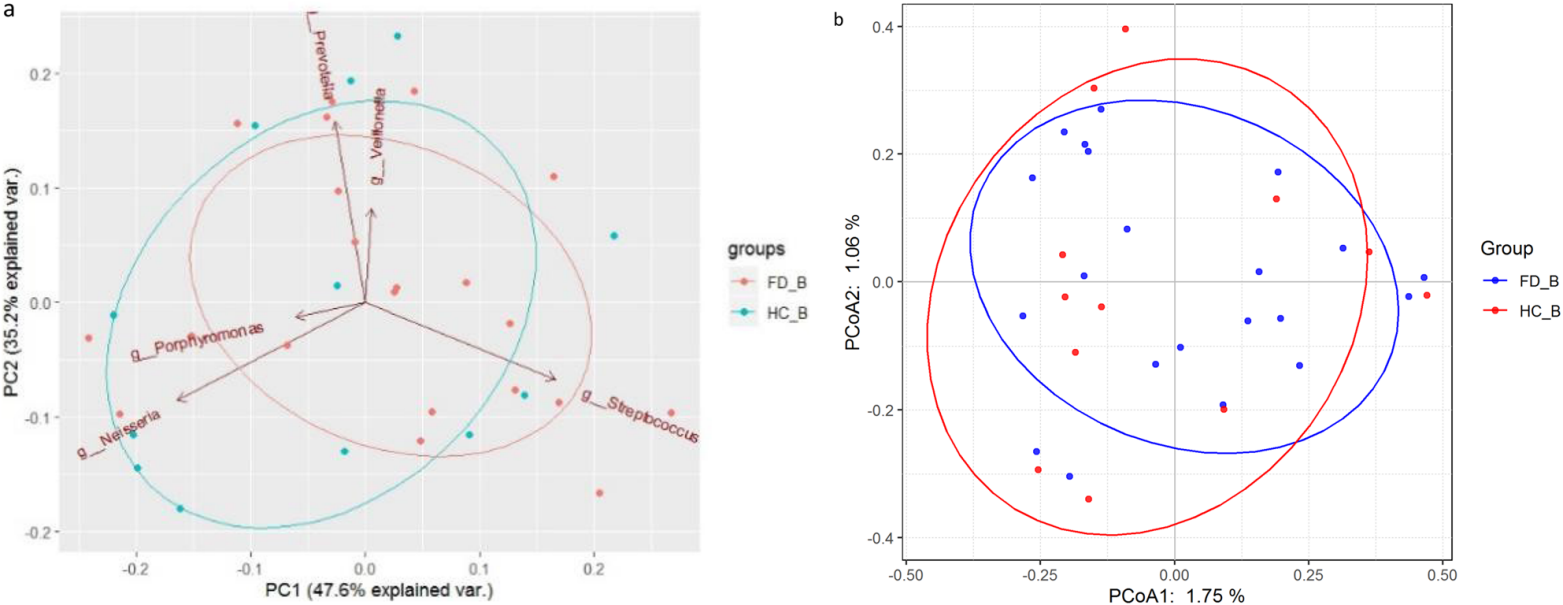
Comparison of the phylogenetic structure and composition of microbial communities between the FD group and HC group. (**a**) Principal Component Analysis (PCA) based on the Bray–Curtis dissimilarity is shown for FD (red) and HC (blue). (**b**) Principal coordinate analysis (PCoA) based on unweighted UniFrac distances is shown for FD (blue) and HC (red).

Bray–Curtis principal component analysis and Bray–Curtis principal coordinate analysis showed that the abundance of oral flora in the HC group was higher than that of the FD group, and there were differences in the phylogenetic structure and composition between the two groups (Fig. 2a, b).

### Saliva biomarkers can be used to classify FD and HC groups

**Figure 3:**
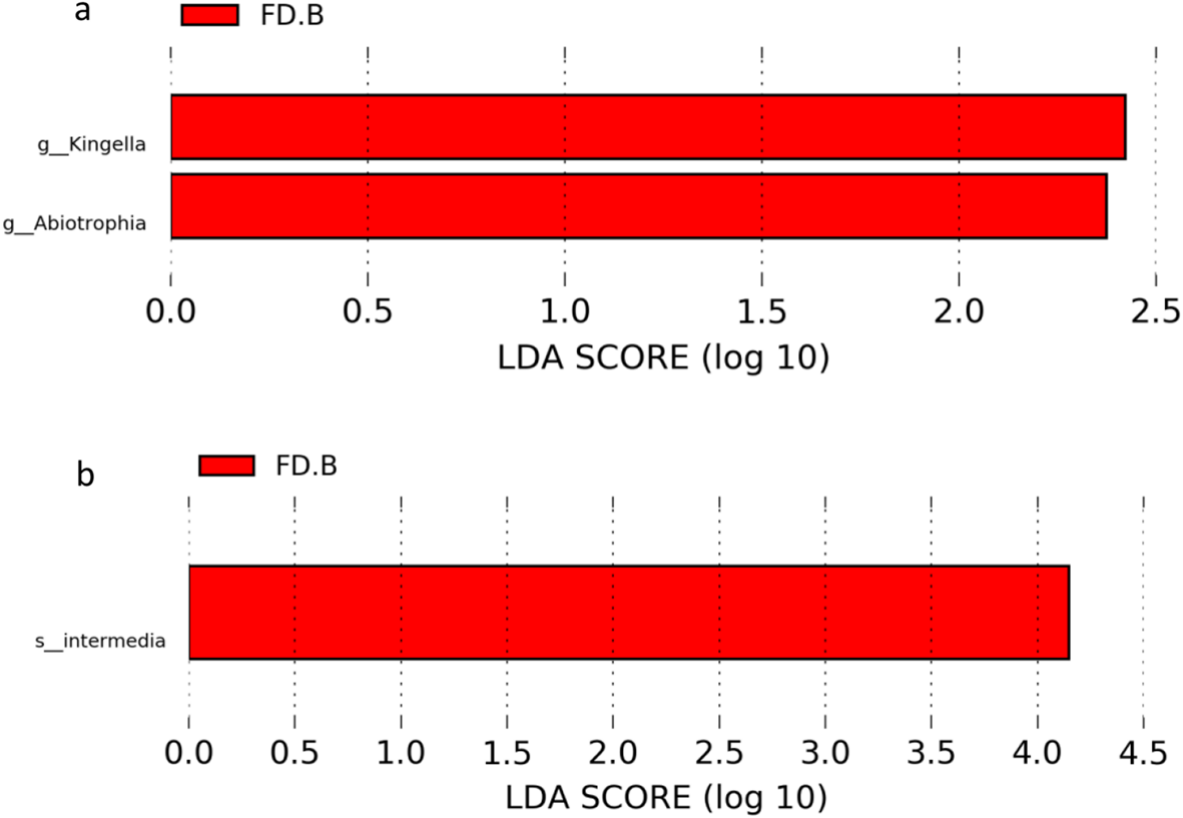
Saliva biomarkers to classify FD and HC groups. (**a**) Saliva genus level LEfSe analysis in FD and HC. (**b**) Saliva microbiota species-level microbiota biomarkers in FD and HC groups. Linear discriminant analysis (LDA) score (less strict: 2; more strict: 4) representing significantly different biomarkers between FD and HC groups. The length of the bar chart represents the significance of the different species.

Further analyses of dysbiosis biomarkers revealed differences in the oral flora of people with FD and in HCs. Genus-level in saliva by LEfSe analysis showed significant changes between the FD group and HCs, including *Kingella* and *Abiotrophia*. Species-level biomarkers showed *Intermedia* to have significant differences between the FD group and HC group (Fig. 3a, b).

### Comparison of salivary pH and CPI between the FD group and HC group

**Figure 4:**
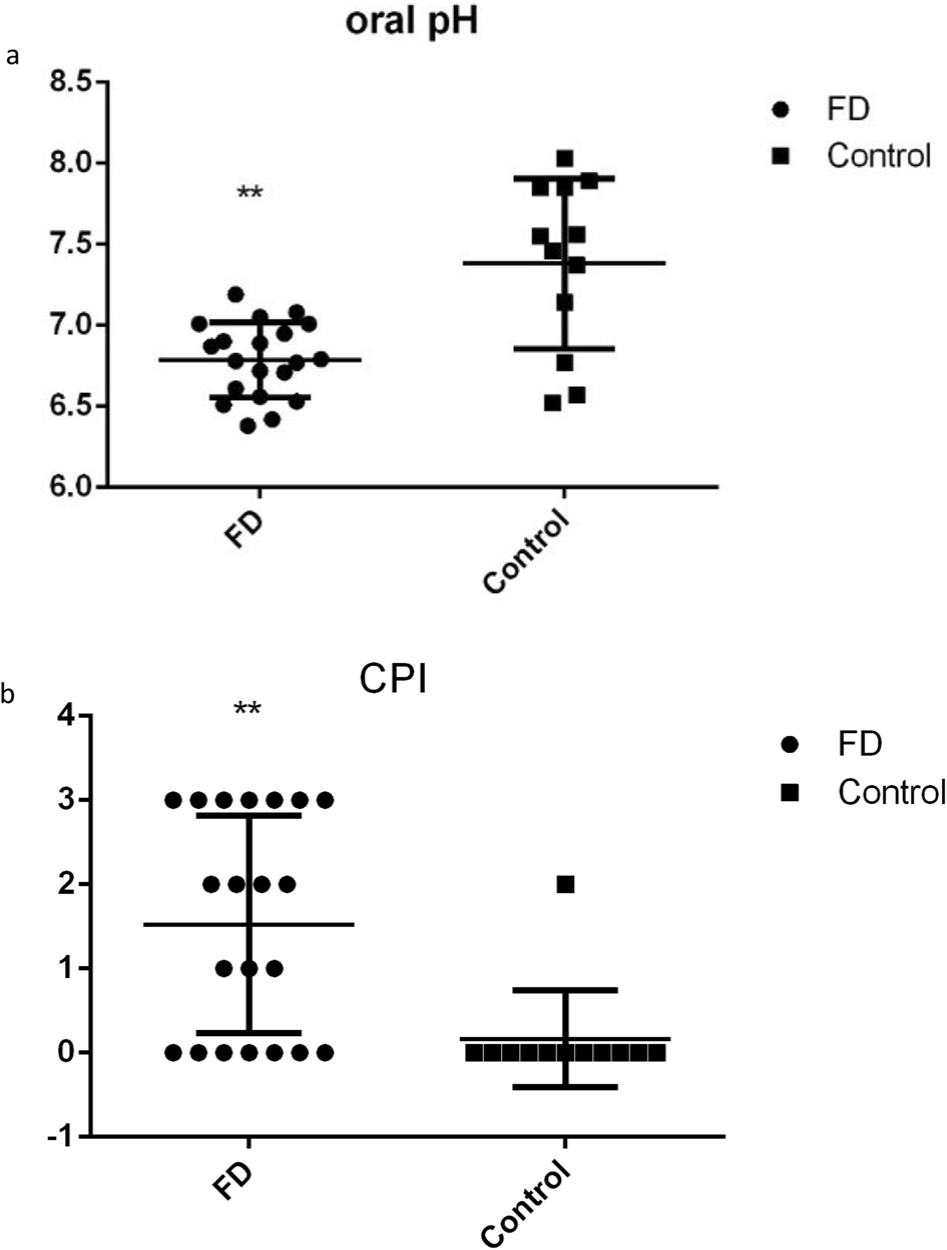
Oral pH and CPI in FD and HC groups. (**a**) Oral pH in FD and HC groups. *p < 0.05; **p < 0.01; (**b**) CPI in FD and HC groups.

Measurement of salivary pH of all participants showed that the mean pH value of the FD group was 6.79 and that of the HC group was 7.48. The oral environment of the FD group was more acidic than that of the HC group (*p* = 0.002) (Fig. 4a). The average CPI of the FD group was 1.52 and that of the HC group was 0.17. The association between the FD group and HC group regarding CPI was significant (*p* = 0.008) (Fig. 4b). DMFT index of FD is 10, DMFT index of HC is 0, there is a significant difference between them (*p* < 0.05).

### SIBO prevalence in FD

**Figure 5:**
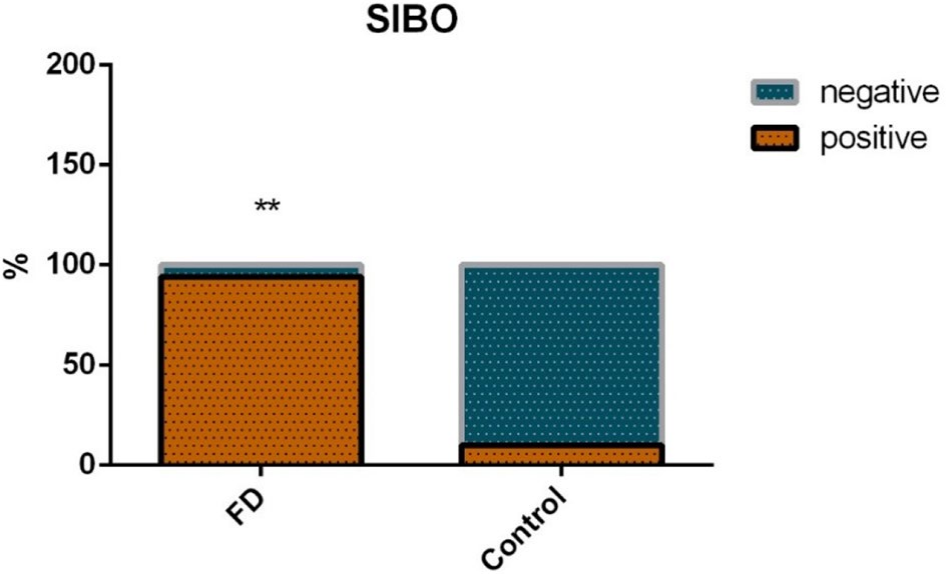
SIBO prevalence in FD and HC groups.

Of the 21 FD patients, 15 (71.4%) had positive results for SIBO according to hydrogen- and methane-based breath tests. In the HC group, only one of 12 (8.3%) patients showed positive results for SIBO (Fig. 5). Significant intergroup differences were noted with respect to SIBO (*p* < 0.05).

### Relationship between salivary pH and microflora in FD

According to the collected oral pH values of patients with FD, they were divided into four parts: 6.38–6.55, 6.56–6.73, 6.74–6.91, and 6.92–7.20, and the correlation between saliva flora and oral pH values of patients with FD was analyzed. Kruskal–Wallis statistics was used to analyze the difference in the abundance of bacteria with different pH levels, and there was no significant statistical difference in the saliva flora (Firmicutes, Proteobacteria, Bacteroidetes, Spirochetes, Actinobacteria, Fusobacteria, Verrucomicrobia, TM7, SR1, GNO2) of FD patients at different oral pH levels (*p* > 0.05), which indicated that the content, abundance and proportion of the saliva flora were similar with different oral pH levels, which indicated that the content, abundance and proportion of the saliva flora were similar with different oral pH levels.

## Discussion

This is the first study to evaluate changes in the oral flora of FD patients using Rome IV criteria.

We showed that the oral flora of people with FD can act as biomarkers. The concentration of genus-level biomarkers in saliva showed significant changes between FD and HC groups, including *Kingella* and *Abiotrophia*. The concentration of species-level biomarkers showed *Intermedia* to have significant differences between FD and HC groups.

Deng et al. showed that changes in the composition of oral flora promotes inflammatory reactions, and leads to oral lichen planus disease^23^. Kitamoto et al.^24^ reported that periodontal inflammation exacerbates gut inflammation in vivo. Vogtmann et al.^25^ found a strong correlation between changes in oral bacteria and the risk of pancreatic cancer. In addition, the intestinal tract and oral cavity share a close relationship and are important components of the human digestive system; moreover, both are major ecological sites of microorganisms in the human body. Nearly all of the saliva produced by adults each day goes into the gastrointestinal tract, so salivary bacteria have good possibilities of colonizing the gut^26^. Sato et al. proposed that orally administered *Porphyromonas gingivalis* (a representative periodontopathic bacterium) alters the gut microbiome^27^. There is a justified association between microbiomes in the mouth and gut in FD, although current evidence that the microbiome causes FD is far from conclusive. Therefore, when exploring FD pathogenesis, the imbalance in oral flora as an important potential mechanism should be considered.

We showed that the mean pH in the mouth of FD patients was 6.78 and that in the HC group was 7.38. The pH in the mouth of FD patients was acidic, whereas that of the HC group was alkaline. Studies have shown that the oral pH of patients with gastroesophageal reflux disease is acidic^28^. However, the correlation between FD and oral pH has not been reported. A low pH in saliva can cause disorders pertaining to oral flora. In addition, a continuous decline in salivary pH can cause further damage to oral soft tissues, leading to ulcers, erythema, oral lichen planus disease, and other diseases of the oral mucosa. In addition, an acidic oral environment induces more acid production by dental plaques, thereby increasing the acidity of the enamel surface and resulting in local decalcification and greater risk of dental caries. We showed that the oral pH of FD cases was more acidic than that of the HCs, which may increase the risk of oral mucosal diseases and dental caries. Therefore, timely detection of oral pH as well as prevention and treatment of an acidic environment are important.

We demonstrated that the CPI and DMFT in the FD group were significantly higher than that in the HC group. This finding suggests that it is easier for patients with FD to develop oral diseases than HCs. Studies have shown that the higher the value of CPI and DMFT, the more prone to oral diseases^29^. FD patients should, therefore, be treated in time to improve oral acidity and reduce the risk of oral diseases.

Consistent with the literature, we demonstrated that SIBO prevalence in FD patients was higher than that in HCs. Costa et al.^30^ showed that SIBO prevalence in FD patients was 56.5%, and that a higher prevalence of SIBO was observed in dyspeptic patients who used proton pump inhibitors compared with that of a control group. Kim et al.^31^ also showed that 2-month treatment with ursodeoxycholic acid resulted in FD-symptom improvement and reduced methane values during 90 min on the lactulose breath test, suggesting that methane-producing SIBO were associated with dyspepsia symptoms. In addition, SIBO is believed to be caused by the lateral movement of intestinal flora, which may trigger a mucosal immune response, leading to the release of cytokines and infiltration of inflammatory cells. This continuous, low-grade inflammatory state changes gastrointestinal motility and permeability, leading to FD onset^32^. The integrity of the duodenal mucosa in FD patients is damaged and permeability is increased, and these structural changes can cause secondary inflammatory reactions and reduce local antibacterial ability, leading to bacterial translocation and SIBO initiation^33^. We hypothesized that a decrease in function of the local mucosal barrier, local inflammatory response, and immune activation in FD patients might cause SIBO.

The main limitation of our study was that the sample size of the study included was small. We will expand the sample size in future studies to verify our data.

## Conclusions

The oral flora of FD patients was different from that of the HCs. Measurement of the genus-level biomarkers *Kingella* and *Abiotrophia* and the species-level biomarker *Intermedia* will be helpful for FD diagnosis. Compared with HCs, patients with FD had a higher prevalence of oral diseases, greater salivary acidity, and higher prevalence of SIBO. These findings are likely to increase awareness of FD pathogenesis in the general population and its detrimental effect upon general health, and will hopefully aid future clinical treatment and guide research planning.
